# Factors associated with plans for early retirement among Ontario family physicians during the COVID-19 pandemic: a cross-sectional study

**DOI:** 10.1186/s12875-024-02374-9

**Published:** 2024-04-18

**Authors:** Rachel Walsh, Deanna Telner, Debra A. Butt, Paul Krueger, Karen Fleming, Sarah MacDonald, Aakriti Pyakurel, Michelle Greiver, Liisa Jaakkimainen

**Affiliations:** 1 Department of Family and Community Medicine, Sunnybrook Health Sciences Centre, Toronto, ON Canada; 2 Department of Family and Community Medicine, University of Toronto, Toronto, ON Canada; 3 Department of Family and Community Medicine, Michael Garron Hospital, Toronto, ON Canada; 4 Department of Family and Community Medicine, Scarborough Health Network, Scarborough, ON Canada; 5 Western University, London, ON Canada; 6 Department of Family and Community Medicine, North York General Hospital, Toronto, ON Canada

**Keywords:** Family physicians, Early Retirement, Job turnover, COVID-19, Pandemic

## Abstract

**Background:**

Higher numbers of family physicians (FPs) stopped practicing or retired during the COVID-19 pandemic, worsening the family doctor shortage in Canada. Our study objective was to determine which factors were associated with FPs’ plans to retire earlier during the COVID-19 pandemic.

**Methods:**

We administered two cross-sectional online surveys to Ontario FPs asking whether they were “planning to retire earlier” as a result of the pandemic during the first and third COVID-19 pandemic waves (Apr-Jun 2020 and Mar-Jul 2021). We used logistic regression to determine which factors were associated with early retirement planning, adjusting for age.

**Results:**

The age-adjusted proportion of FP respondents planning to retire earlier was 8.2% (of 393) in the first-wave and 20.5% (of 454) in the third-wave. Planning for earlier retirement during the third-wave was associated with age over 50 years (50–59 years odds ratio (OR) 5.37 (95% confidence interval (CI):2.33–12.31), 60 years and above OR 4.18 (95% CI: 1.90-10.23)), having difficulty handling increased non-clinical responsibilities (OR 2.95 (95% CI: 1.79–4.94)), feeling unsupported to work virtually (OR 1.96 (95% CI: 1.19–3.23)) or in-person (OR 2.70 (95% CI: 1.67–4.55)), feeling unable to provide good care (OR 1.82 (95% CI: 1.10–3.03)), feeling work was not valued (OR 1.92 (95% CI: 1.15–3.23)), feeling frightened of dealing with COVID-19 (OR 2.01 (95% CI: 1.19–3.38)), caring for an elderly relative (OR 2.36 (95% CI: 1.69–3.97)), having difficulty obtaining personal protective equipment (OR 2.00 (95% CI: 1.16–3.43)) or difficulty implementing infection control practices in clinic (OR 2.10 (95% CI: 1.12–3.89)).

**Conclusions:**

Over 20% of Ontario FP respondents were considering retiring earlier by the third-wave of the COVID-19 pandemic. Supporting FPs in their clinical and non-clinical roles, such that they feel able to provide good care and that their work is valued, reducing non-clinical (e.g., administrative) responsibilities, dealing with pandemic-related fears, and supporting infection control practices and personal protective equipment acquisition in clinic, particularly in those aged 50 years or older may help increase family physician retention during future pandemics.

**Supplementary Information:**

The online version contains supplementary material available at 10.1186/s12875-024-02374-9.

## Background

The COVID-19 pandemic added considerable strain to the workload and personal lives of family physicians (FPs) in Ontario. FPs provide the majority of primary care in Ontario, which is integral in providing healthcare to Ontarians [1], and contributes to better health outcomes, improved health equity, and lower costs in health systems worldwide [2, 3]. The COVID-19 pandemic resulted in FPs working more hours with increased levels of work stress and burnout [4, 5]. This was exacerbated by low preparedness, high work and personal life impact of the pandemic, and fears of contracting COVID-19, especially among older FPs with comorbidities [6–8].

During the pandemic, we saw large numbers of FPs retiring, worsening the pre-existing shortage of FPs in Ontario and Canada [9, 10]. The rate of Ontario FPs who stopped practicing doubled in the first 6 months of the pandemic [11]. During the first-wave of the COVID-19 pandemic, almost 1.8 million Ontarians already did not have a regular FP. This number is expected to increase to 3 million Ontarians by the year 2025, with retirement of existing FPs being a main driver [9, 12].

Knowing which factors influenced early retirement of FPs in Ontario during the COVID-19 pandemic can allow us to identify areas for improvement to help ensure increased retention of the primary care workforce during future pandemics. Previous studies have found that intent to change jobs/leave clinical care (turnover intention) among various health care professionals predicts actually leaving clinical care [13]. A systematic review of healthcare workers’ turnover intentions during the COVID-19 pandemic found forty-three studies of nurses, physicians, and other healthcare workers, but none examining FPs specifically. This review identified fear of COVID-19 exposure (including increased risk due to insufficient infection prevention and control (IP&C) measures), psychological responses to stress (including anxiety, depression, burnout, psychosocial issues, low professional commitment, low job satisfaction, and poor resilience), socio-demographic characteristics (e.g., poor social or peer support, being married, male, and both older and younger), adverse working conditions (e.g., increased workload, work hours and work effort, workplace violence, changes at work, deployment to other departments, poor job resources, low staff morale), and organizational support (including poor employer communication and job preparedness, poor organizational atmosphere and motivation, and low rewards from work) as five themes associated with turnover intention [14]. While none of the studies included in the systematic review looked specifically at FPs, one Ontario-based study from Toronto found that older male FPs caring for more patients (an additional 166 patients on average) in solo practice were more likely to consider early retirement during the COVID-19 pandemic [15]. It is unclear how many of the other workplace factors associated with turnover intention among healthcare workers in general have contributed to the increase in Ontario FPs retiring during the COVID-19 pandemic. Some factors suggested by the Ontario COVID-19 science table to be contributing to increased Ontario FPs leaving practice include lack of team-based supports, administrative and operational challenges, and unpredictability of fee-for-service compensation models [12]. These factors were not identified as being related to turnover intention among healthcare workers in the systematic review. It is possible that these factors are more contextually relevant to FPs in Ontario.

Consequently, the objective of our current paper was to determine how previously identified factors from the literature (i.e., pandemic-related fears, psychological responses to stress, socio-demographic characteristics, adverse working conditions, organizational support) affected the intentions of Ontario FPs to retire earlier during the COVID-19 pandemic, as well as how additionally important local factors (i.e., team-based models, non-clinical workload, and compensation model) affected Ontario FPs’ desires to retire earlier during the COVID-19 pandemic.

## Methods

### Study aims

We aimed to identify which factors affected FPs’ desires to retire earlier during the COVID-19 pandemic. Based on previous literature, we hypothesized that early retirement planning would be associated with COVID-19-related fears (including difficulties with IP&C practices, and having comorbidities or a relative with comorbidities), psychological factors (negative personal life impacts from the pandemic, feeling a duty to provide care), socio-demographic characteristics (age, gender), work conditions (work satisfaction, solo versus group practice, compensation model, clinical and non-clinical workload, working in team-based models), and organizational support (including feeling work was valued). We additionally aimed to create a shortlist of which factors might be most important for Ontario FP turnover during the pandemic.

### Participants and design

We distributed two cross-sectional online surveys to actively practicing Ontario FPs. Our first survey was open from October 5, 2020 to February 26, 2021 and asked about experiences during the first-wave of the COVID-19 pandemic (April-June 2020). Our second survey was open from May 21 to August 2, 2021 and asked about experiences during the third-wave of the COVID-19 pandemic (March-July 2021) [16]. Medical learners and residents were excluded from participating in the survey.

### Setting and survey distribution

We distributed our first-wave survey through email lists of 39 Ontario Health Teams (OHTs; cooperative groups of Ontario healthcare providers and organizations) [17] and 4 Toronto-based primary care teams, reaching 1,726 physicians, and on 3 online forums/Facebook groups targeted towards Canadian/Ontarian FPs. We distributed our third-wave survey through newsletters from the Section of General and Family Practice (SGFP) of the Ontario Medical Association (OMA) as well as through posts on their official website. The SGFP represents approximately 15,000 FPs in Ontario [18]. In order to achieve a 5% margin of error for our survey responses, we aimed to receive at least 375 survey responses during each wave.

### Measurements

The survey was pre-tested amongst the research team, including content and methodology experts, and potential FP respondents. We piloted the survey, formatted using Qualtrics software [19], with 13 FPs in the Sunnybrook Academic Family Health Team in Toronto, Ontario. The final survey contained 42 items and explored physician willingness and perceived support to provide care during the COVID-19 pandemic, as well as clinical, personal, and demographic factors (see Additional File 1 for the third-wave survey questions). This survey was adapted from a survey administered to FPs in the Greater Toronto Area (GTA) after the Severe Acute Respiratory Syndrome (SARS) outbreak of 2003 and H1N1 influenza outbreak of 2009 [20]. In our current survey, we asked FP respondents to indicate if they “plan to retire earlier” as a result of the COVID-19 pandemic. Those who selected this option were compared to those who did not as the primary outcome for this study. While the survey’s original main outcome was willingness and support to provide care during the pandemic, planning to retire earlier became the focus for this analysis due to system-wide concerns about the growing numbers of patients without a FP.

Age information was collected categorically as per the previous SARS and H1N1 surveys. FP respondents were deemed to work in specialized/focused practice if they indicated they worked in any area besides general family practice or walk-in clinic, which could be instead of, or in addition to, their work in these settings. Non-clinical responsibilities were not specifically defined in the survey, and would include administrative, teaching, research, or other activities not directly related to patient care. Survey items collected on a 5-point Likert scale were dichotomized into positive and negative/neutral responses (e.g., Agree/Strongly Agree vs. Strongly Disagree/Disagree/Neutral). The decision to dichotomize Likert scale items was made a priori for ease of presentation and analysis of results. The decision to group neutral responses with negative response categories (e.g. Neutral/Disagree/Strongly Disagree) was also made a priori, before examining the responses.

### Statistical analysis

Data from partially completed surveys were included in our analysis. We calculated descriptive statistics for all survey questions. Survey items with missing responses were removed from the denominator for that question. In order to account for age distribution differences between the first- and third-wave surveys, age-adjusted rates of our primary outcome were calculated using weights from the Canadian Institute for Health Information [21]. We used the chi-squared test, or when appropriate, Fisher’s exact test to compare survey responses to our primary outcome. We used logistic regression to calculate odds ratios for factors associated with earlier retirement planning, adjusting for age. We performed multivariable logistic regression using stepwise regression for both the first- and third-wave surveys. To avoid multicollinearity, we excluded variables with variance inflation factors > 10. To avoid overfitting the models, we limited the number of model variables to 1/10th the number of observations. We calculated Hosmer-Lemeshow goodness-of-fit *p*-values and C-statistics for the multivariable models. A *p* value of < 0.05 with an odds ratio 95% confidence interval that did not cross 1.0 was considered statistically significant. All data analyses were performed in R (version 4.0.3) using the glmtoolbox (v0.1.4) [22], DescTools (v.0.99.44) [23] and leaps (v3.1) [24] packages.

## Results

There were 393/458 eligible responses to our first-wave survey, and 454/470 eligible responses to our third-wave survey, with an undetermined proportion responding to both surveys (Table 1). This resulted in a margin of error of 4.9% for the first-wave survey and 4.5% for the third-wave survey. Compared to the third-wave survey, the first-wave survey had more respondents under 39-years of age (51.2% vs. 21.0%), practicing in rural communities with populations under 10,000 (16.9% vs. 7.9%), practicing in larger group practices (64.5% vs. 44.6% in groups with over 5 physicians), being paid by capitation (63.1% vs. 55.1%), and teaching residents (40.9% vs. 30.8%). In the third-wave survey, there were more respondents being paid by fee-for-service (FFS; 32.5% vs. 19.1%). The proportion of team-affiliated FPs was similar across both surveys (37.0% working in an OHT in the first-wave vs. 31.8% third-wave; 77.9% working with allied health in the first-wave vs. 72.1% third-wave) (Table 1).

**Table 1:** Demographics and practice characteristics of ontario FP respondents during the first and third pandemic waves

Demographic and practice characteristics (n 1st wave; n 3rd wave)	Pandemic wave	
	First wave (April-June 2020)	Third wave (March-July 2021)
	Total (*n* = 393)	Total (*n* = 454)
	n (%)	n (%)
**Planning to retire earlier due to the COVID-19 pandemic** (*n* = 393; *n* = 454)	24 (6.1%)	94 (20.7%)
**Age** (*n* = 367; *n* = 420)		
Under 39	188 (51.2%)	88 (21.0%)
40–49	73 (19.9%)	104 (24.8%)
50–59	63 (17.2%)	116 (27.6%)
60 or over	43 (11.7%)	112 (26.7%)
**Gender** (*n* = 367; *n* = 420)		
Female	244 (66.5%)	276 (65.7%)
Male	118–123 (32.2–33.5%)	144 (34.3%)
Other/non-binary	< 5 (< 1.4%)	
**Community size (population)** (*n* = 360; *n* = 420)		
Rural (< 10,000)	61 (16.9%)	33 (7.9%)
Urban (10,000–99,999)	62 (17.2%)	78 (18.6%)
Urban (100,000-999,999)	80 (22.2%)	148 (35.2%)
Urban (> 1,000,000)	157 (43.6%)	161 (38.3%)
**GTA** (*n* = 367; *n* = 420)	181 (49.3%)	210 (50.0%)
**Specialized/focused practice** (*n* = 367; *n* = 420)	215 (60.2%)	213 (50.7%)
**Any allied health available through office/practice** (*n* = 393; *n* = 454)	306 (77.9%)	328 (72.1%)
**Number of physicians in practice** (*n* = 358; *n* = 412)		
1	39 (10.9%)	83 (20.1%)
2	22 (6.1%)	50 (12.1%)
3–4	66 (18.4%)	95 (23.1%)
5–9	151 (42.2%)	108 (26.2%)
10+	80 (22.3%)	76 (18.4%)
**Payment model** (*n* = 366; *n* = 419)		
Fee-for-service	70 (19.1%)	136 (32.5%)
Capitation	231 (63.1%)	231 (55.1%)
Hourly/Sessional fee	10 (2.7%)	13 (3.1%)
Other	27 (7.4%)	17 (4.1%)
Salary	28 (7.7%)	22 (5.3%)
**Working fulltime pre-pandemic** (*n* = 367; *n* = 419)	308 (83.9%)	350 (83.5%)
**Works with residents** (*n* = 367; *n* = 419)	150 (40.9%)	129 (30.8%)
**Works in an OHT** (*n* = 365; *n* = 418)	135 (37.0%)	133 (31.8%)
**Uses EMR** (*n* = 367; *n* = 420)	350 (97.8%)	386 (91.9%)

The age-adjusted proportion of Ontario FPs planning to retire earlier due to the COVID-19 pandemic was 8.2% in the first-wave and 20.5% in the third-wave. Older FPs were more likely to plan for early retirement compared to those under age 39. There was no significant association with physician gender (Table 2). The proportion of respondents planning to retire earlier compared to each survey item is available in Additional File 2.

**Table 2:** Unadjusted odds ratios for physician age and gender associated with planning to retire early

**Demographic Characteristic**	Pandemic wave			
	First wave		Third wave	
**Age**	**Odds ratio (95% CI)**	P value	**Odds ratio (95% CI)**	P value
*Under 39*	reference	reference		
*40–49*	2.32 [0.72,7.21]	0.144	1.43 [0.57,3.77]	0.452
*50–59*	2.23 [0.64,7.25]	0.185	5.07 [2.33,12.31]	< 0.001*
*60 or over*	4.19 [1.28,13.34]	0.014*	4.18 [1.90,10.23]	0.001*
**Male gender**	1.77 [0.76,4.10]	0.177	1.01 [0.62,1.63]	0.977

*p < 0.05

CI = confidence interval

In both waves, after adjusting for age, earlier retirement planning was associated with feeling unable to handle work or specifically increased non-clinical responsibilities, and feeling unsupported to work virtually (Table 3). In the first-wave, after adjusting for age, earlier retirement planning was additionally associated with not having an electronic medical record (EMR), working in practices with < 3 physicians, feeling unsafe travelling to clinic, and being unwilling to work in-person. In the third-wave, after adjusting for age, early retirement planning was additionally associated with caring for an elderly relative, feeling frightened of dealing with COVID-19, not feeling a duty to provide care during the pandemic, feeling unable to handle personal responsibilities, feeling unable to provide good clinical care, feeling work during the pandemic was not valued, feeling unsupported to work in-person, or feeling that IP&C practices in clinic, obtaining PPE, or managing staff/personal fear needed improvement.

The following factors were not associated with early retirement planning in either the first- or third-waves: willingness to work virtually, training/experience with infectious disease outbreaks, worries about family contracting COVID-19, satisfaction with public health measures, changing income or work hours, family or personal pregnancy or comorbidities, being a parent, urban/rural or GTA location, practicing in a focused area, or with allied health, payment model, working part-time, with residents, or within an OHT, or needing support with isolation rooms, clinic leadership, COVID-19 testing, COVID-19 cases, PPE use, or physical distancing (Table 3). See Additional File 2 for unadjusted survey responses.

**Table 3:** Age-adjusted odds ratios for early retirement planning during the first and third pandemic waves

Demographic and practice characteristics	First wave (April-June 2020)		Third wave (March-July 2021)	
	Age-adjusted OR [95% CI]	p value	Age-adjusted OR [95% CI]	p value
**Willing to work in-person**	0.28 [0.12,0.67]	0.004*	0.56 [0.27,1.20]	0.122
**Willing to work virtually**	0.96 [0.25,6.29]	0.957	1.04 [0.43,2.77]	0.941
**Supported to work in-person**	0.38 [0.12,0.98]	0.061	0.37 [0.22,0.60]	< 0.001*
**Supported to work virtually**	0.39 [0.15,0.91]	0.034*	0.51 [0.31,0.84]	0.008*
**Needed improvement**				
*Isolation room in clinic*	0.99 [0.35,2.50]	0.989	1.72 [0.97,3.01]	0.058
*Clinic leadership*	1.52 [0.53,3.85]	0.399	1.79 [0.93,3.36]	0.075
*Managing COVID testing*	1.48 [0.63,3.44]	0.360	1.08 [0.58,1.92]	0.809
*Managing potential COVID cases*	1.23 [0.52,2.85]	0.634	1.01 [0.58,1.71]	0.971
*Using PPE appropriately*	0.80 [0.26,2.11]	0.677	1.90 [0.86,4.07]	0.103
*Managing staff/personal fear*	1.42 [0.61,3.40]	0.418	2.16 [1.28,3.64]	0.004*
*Obtaining PPE*	0.83 [0.35,2.06]	0.670	2.00 [1.16,3.43]	0.012*
*Physical distancing in clinic*	1.57 [0.66,3.69]	0.298	1.16 [0.65,2.02]	0.612
*Infection control practices in clinic*	1.01 [0.43,2.37]	0.976	2.10 [1.12,3.89]	0.019*
*Handling increased non-clinical responsibilities*	4.27 [1.75,11.54]	0.002*	2.95 [1.79,4.94]	< 0.001*
**Felt safe travelling to clinic**	0.19 [0.08,0.49]	< 0.001*	0.57 [0.29,1.17]	0.113
**Training in infectious disease outbreaks**	1.39 [0.43,3.80]	0.550	0.60 [0.28,1.19]	0.166
**Experience with infectious disease outbreaks**	1.12 [0.43,2.96]	0.820	1.02 [0.60,1.74]	0.939
**Frightened of Dealing with COVID-19**	2.69 [1.03,8.39]	0.058	2.01 [1.19,3.38]	0.009*
**Worried about infecting family**	2.24 [0.61,14.44]	0.294	1.35 [0.81,2.29]	0.252
**Family worried about getting infected**	1.77 [0.67,5.55]	0.281	1.23 [0.75,2.01]	0.409
**Felt able to provide good clinical care**	1.22 [0.49,3.48]	0.689	0.55 [0.33,0.91]	0.037*
**Work during the pandemic was valued**	1.01 [0.39,2.92]	0.992	0.52 [0.31,0.87]	0.011*
**Satisfied with ability to handle work responsibilities during the pandemic**	0.32 [0.13,0.76]	0.010*	0.27 [0.16,0.46]	< 0.001*
**Satisfied with ability to handle personal responsibilities during the pandemic**	1.24 [0.52,3.18]	0.639	0.59 [0.35,0.97]	0.038*
**Duty to provide care during the pandemic**	0.22 [0.06,1.07]	0.034*	0.20 [0.07,0.61]	0.004*
**Satisfied with public health measures to prevent community spread**	0.43 [0.15,1.07]	0.084	0.64 [0.39,1.04]	0.072
**Lost Income**	1.31 [0.50,3.84]	0.600	1.35 [0.79,2.32]	0.279
**Decreased hours**	2.16 [0.88,5.10]	0.084	1.32 [0.75,2.29]	0.321
**Increased hours**	0.54 [0.15,1.49]	0.279	0.84 [0.50,1.38]	0.486
**Pregnancy (You or spouse)**	2.01 [0.28,9.83]	0.416	0.45 [0.02,2.48]	0.452
**Personal medical conditions**	1.76 [0.64,4.51]	0.250	1.44 [0.83,2.44]	0.186
**Family member with medical conditions**	1.56 [0.62,3.72]	0.320	1.08 [0.66,1.76]	0.757
**Parent**	0.94 [0.36,2.58]	0.907	0.89 [0.52,1.54]	0.680
**Care for elderly relative**	1.46 [0.39,4.30]	0.528	2.36 [1.39,3.97]	0.001*
**Community size (population)**				
*Rural (< 10k)*	0.43 [0.07,1.69]	0.288	1.43 [0.56,3.40]	0.434
*Urban (10-100k)*	0.72 [0.19,2.19]	0.590	0.85 [0.41,1.70]	0.650
*Urban (100k-1M)*	1.19 [0.39,3.31]	0.752	1.13 [0.65,1.97]	0.669
*Urban (> 1M)*	reference		reference	
**Practices outside the GTA**	0.55 [0.22,1.27]	0.169	1.04 [0.65,1.68]	0.859
**Specialized/focused practice**	0.55 [0.23,1.28]	0.162	0.74 [0.46,1.20]	0.227
**Any allied health available through office/practice**	0.40 [0.16,1.10]	0.060	0.66 [0.38,1.14]	0.128
**Number of Physicians in Practice**				
*1*	reference		reference	
*2*	0.39 [0.05,1.85]	0.274	1.87 [0.85,4.13]	0.121
*3–4*	0.07 [0.00,0.44]	0.017*	1.07 [0.50,2.30]	0.853
*5–9*	0.25 [0.08,0.80]	0.018*	0.75 [0.35,1.57]	0.440
*10+*	0.28 [0.08,0.95]	0.044*	1.17 [0.54,2.55]	0.688
**Payment Model**				
*Fee-for-service*	reference		reference	
*Capitation*	1.21 [0.42,4.42]	0.744	0.81 [0.49,1.37]	0.432
*Hourly/Sessional fee*	2.86 [0.13,24.27]	0.385	0.46 [0.02,2.63]	0.468
*Salary*	2.22 [0.40,11.18]	0.328	1.22 [0.39,3.46]	0.720
**Working Part-time pre-pandemic**	2.32 [0.84,5.81]	0.084	0.80 [0.40,1.51]	0.498
**Works with Residents**	0.80 [0.32,1.88]	0.617	0.87 [0.49,1.50]	0.612
**Works in an OHT**	0.46 [0.16,1.16]	0.118	0.86 [0.50,1.43]	0.555
**Does not use an EMR**	6.57 [1.20,30.71]	0.019*	1.49 [0.66,3.22]	0.322

*p < 0.05

OR = odds ratio; CI = confidence interval

PPE = personal protective equipment’; GTA = Greater Toronto Area; OHT = Ontario Health Team; EMR = Electronic Medical Record

Our first-wave multivariable model found that early retirement planning was associated with not using EMR, and feeling unsafe travelling to clinic (Table 4). This model had a C-statistic of 0.68, a Hosmer-Lemeshow *p*-value of 0.90, and excluded 28 respondents due to missing values. Our third-wave multivariable model found that early retirement planning was associated with being 50 years of age or older, feeling unable to handle increased non-clinical or work responsibilities, feeling unsupported to work in-person, providing care for an elderly relative, and not feeling a duty to work during the pandemic. This model had a C-statistic of 0.79, a Hosmer-Lemeshow *p*-value of 0.06, and excluded 41 respondents due to missing values.

**Table 4:** Odds ratios for early retirement planning during the first and third pandemic waves, multivariable models

First wave (April-June 2020)*	**OR [95% CI]**	***p *** **value**
Does not use EMR	9.57 [1.75,44.85]	0.005
Did not feel safe travelling to clinic	4.15 [1.67,10.03]	0.002
Third wave (March-July 2021)*	**OR [95% CI]**	***P *** **value**
Satisfied with ability to handle work responsibilities	0.39 [0.22,0.69]	0.001
Age under 39	reference	
Age 40–49	1.71 [0.62,5.03]	0.305
Age 50–59	7.57 [3.11,20.85]	< 0.001
Age 60 or over	7.81 [3.14,21.89]	< 0.001
Felt that ability to handle non-clinical responsibilities needed improvement	2.09 [1.19,3.67]	0.010
Felt supported to work in-person	0.54 [0.31,0.94]	0.030
Felt a duty to work during the pandemic	0.30 [0.09,0.95]	0.038
Felt that PPE use needed improvement	1.89 [0.78,4.40]	0.146
Providing care for an elderly relative	1.83 [1.02,3.26]	0.041

*Results adjusted for all other variables included in model

## Discussion

This study adds valuable data concerning earlier retirement plans among Ontario FPs during the COVID-19 pandemic. The proportion of FP respondents planning to retire earlier was 2.5-fold higher in the third-wave survey compared to the first-wave survey (20.5% vs. 8.2%). Planning for retirement was higher in a younger cohort (those aged 50 to 59) in the third-wave survey, potentially implying a pending worsening of the health human resource crisis in primary care. In Ontario, 3.1% of practicing FPs stopped working during the first 6 months of the COVID-19 pandemic – double that from previous years [11]. Our work indicates that this proportion likely continued to increase throughout the pandemic.

Our study identified several important factors that may be contributing to earlier retirement of Ontario FPs during the COVID-19 pandemic. Consistent with previous literature [14], we identified that COVID-19-related fears (including trouble maintaining IP&C practices and procuring PPE, and caring for an elderly relative), psychological factors (not feeling a duty to provide care, being unwilling to work in-person, feeling unable to handle personal responsibilities), sociodemographic characteristics (older age), work conditions (feeling unable to handle work and specifically non-clinical responsibilities, feeling unable to provide good care, working in practices with < 3 physicians), and organizational support (feeling unsupported to work in-person and virtually, feeling that work was not valued) were related to Ontario FPs’ intentions to retire earlier during the COVID-19 pandemic. Interestingly, having a comorbidity or relative with comorbidity, physician payment model, and working in teams with allied health were not associated with planning to retire earlier. In our study, increased non-clinical responsibilities were a significant concern for FP respondents. While, non-clinical responsibilities encompass many activities not directly related to patient care, the administrative workload of FPs has been most widely deemed a contributing factor to FP burnout [10, 25, 26]. While a recent science brief from the COVID-19 science table suggested that a lack of team-based supports, administrative and operational challenges, and unpredictability of fee-for-service compensation models may be contributing to the health human resource crisis in Ontario [12], our data suggests that the administrative and operational challenges likely play a larger role in FPs retiring early, rather than lack of team-based supports or compensation model.

The results of our multivariable analyses highlight the importance of COVID-19-related fears (feeling safe travelling to clinic) and working conditions (specifically EMR use, likely indicating facility transitioning to virtual care that occurred during the first-wave) [27, 28] during the first-wave. It also highlights the importance of all themes (COVID-19 fears/caring for elderly relative, psychological/duty to provide care, sociodemographic/age, working conditions/handling work and non-clinical responsibilities, and organizational support/to work in-person) during the third-wave. While our multivariable analyses are limited by the number of included variables (done in order to avoid overfitting) as well as the limitations of step-wise regression [29], these results can help guide future work by encouraging these factors to be specifically considered when examining turnover intention of FPs during times of stress.

This study has several strengths. Firstly, we were able to explore a large, comprehensive set of factors identified in the literature that were associated with early retirement planning among FPs in the primary care setting. Secondly, we were able to include factors identified specifically in the Ontario context, which can help shed light on questions raised locally to help with provincial resource planning and health policy. Thirdly, we received a large number of responses, resulting in a small margin of error, allowing confidence in our effect estimates within the responding population.

The results of our study should be interpreted in light of a few potential limitations. Firstly, due to the use of social media/online forums in our first-wave survey, with the invitation to participate viewed by an unknown number of eligible respondents, we were unable to calculate an accurate response rate. At best, our first-wave survey had a response rate of 26.5% if including only the OHT email lists in the denominator. For our third-wave survey, the SGFP of the OMA was unable to provide an exact number of eligible up-to-date email addresses included in their listserv. Therefore, at worst, if all ∼ 15,000 Ontario FPs were effectively reached by the monthly SGFP newsletter email, our response rate would be 3.1%. As such, it is important to recognize potential nonresponse bias, such that those who responded may have had more extreme opinions or differed in other ways from those that did not respond. Secondly, our respondents differed from the general population of Ontario FPs in a few areas. Approximately half of our first-wave survey respondents were under 39-years-old. This was likely due to our first-wave distribution strategy involving online forums/Facebook groups directed towards younger physicians. We accounted for this in our analyses by determining age-adjusted rates and odds ratios. Our survey had a greater proportion of female respondents compared to the overall Canadian FP population (65.7–66.5% female in our survey vs. 47.0% of Canadian FPs), as well as a smaller proportion of respondents being paid by FFS (19.1–32.5% vs. 44.0% of Canadian FPs) [18]. Therefore, our survey may be overrepresenting the opinions of younger FPs in the first-wave, and underrepresenting the opinions of male FPs being paid through FFS compared to the general FP population. Thirdly, we did not correct for multiple comparisons in our study, increasing the likelihood of type I error. Fourthly, the data reported are from two different samples/surveys, one from the first wave and the second from the third wave. The sampling methods were different between surveys and resulted in samples that differed in age distribution and clinical specialties. Generalizations and direct comparisons made between the two samples are therefore limited. However, we note that our findings are consistent with other studies showing increased, but relatively lower rates of retirement earlier in the COVID-19 pandemic (3.1% of Ontario family physicians retiring during the first wave) and higher rates of plans for earlier retirement later in the pandemic (17.5% of Toronto family physicians during the third wave planning to close their existing practices in the next 5 years) [11, 15]. Fifthly, due to delays in the research process and difficulty predicting the timing of COVID-19 waves, the distribution of our surveys did not always coincide with the COVID-19 wave in question. Respondents were therefore asked, for the most part, to reflect on the previous COVID-19 wave after it had passed. Although we specified in the survey to reflect on experiences during the previous COVID-19 wave, it is possible that responses were influenced by post-wave perceptions.

The results of this study can help guide future policy and research to alleviate and prevent worsening of the current family medicine crisis in Canada [9, 30]. We identified modifiable factors relevant to health organizations and policy-makers that may increase FP retention during future pandemics. Specifically, improved retention of Ontario FPs during future pandemics may be attainable if FPs feel supported in their clinical and non-clinical roles, such that they feel able to provide good care, and that their work is valued. Pandemic-specific modifiable factors included managing pandemic-related fears, and supporting IP&C practices and PPE procurement for FPs. Additional retention of FPs working during the pandemic may be achieved by motivating FPs through their duty to provide care, targeting physicians over 50-years-old, and helping support FPs caring for elderly relatives. Earlier retirement of FPs during future pandemics may be mitigated by encouraging FPs to work in larger group practices. Future work from our group will look further at this survey data to identify factors related to feeling willing and supported to work during the pandemic. Other avenues for future research include determining what, including both pecuniary and non-pecuniary factors, makes FPs feel valued in their work, clarifying why caring for an elderly relative, but not necessarily one with a COVID-susceptible comorbid illness, was associated with wanting to retire earlier during the COVID-19 pandemic, how best to manage pandemic-related fears among FPs, and whether the increases in FP retirement throughout the COVID-19 pandemic predicted by our data actually occurred.

## Conclusions

In conclusion, our study showed that over 1 in 5 FPs were considering retiring earlier during the third-wave of the COVID-19 pandemic. Providing support to FPs in their clinical and non-clinical roles, creating an environment where they feel valued and able to provide good clinical care, reducing non-clinical (e.g., administrative) responsibilities, managing pandemic-related fears, and supporting IP&C practices in clinic and PPE procurement may help reduce the number of FPs retiring prematurely during future pandemics.

### Electronic supplementary material

Below is the link to the electronic supplementary material.

Supplementary Material 1

Supplementary Material 2

## Data Availability

The datasets used and/or analysed during the current study are available from the corresponding author on reasonable request.

## Abbreviations

CI: Confidence interval
COVID-19: Coronavirus disease of 2019
EMR: Electronic medical record
FFS: Fee-for-service
FP: Family physician
GTA: Greater Toronto Area
IP&C: Infection prevention and control
OHT: Ontario Health Team
OMA: Ontario Medical Association
OR: Odds ratio
PPE: Personal protective equipment
SGFP: Section of General and Family Practice
