# Supplementary material for: Factors associated with plans for early retirement among Ontario family physicians during the COVID-19 pandemic: a cross-sectional study
Source: BMC Prim Care. 2024 Apr 18;25:118. doi: 10.1186/s12875-024-02374-9 (PMC11025226; doi:10.1186/s12875-024-02374-9)
Supplement: Supplementary file 1 — Supplementary Material 1 [file 12875_2024_2374_MOESM1_ESM.docx]

**Supporting information**

**S1. Third-wave survey questions. The survey was distributed online using Qualtrics software (Qualtrics, Provo, UT, 2021)**

WILLINGNESS to provide care during the COVID-19 pandemic third wave
*Please answer the following questions in relation to your experience during March 2021 to present, the third wave of the COVID-19 epidemic. Please answer questions as they pertain to your existing* ***main*** primary care practice.

**Q1. Are you a primary care physician who has provided care in Ontario at any point since March 2020?** (Select one response)

- Yes
- No

**Q3. Are you a resident or medical trainee?**

- Yes
- No

Q4a. How willing are you to provide *in-person* clinical care during the third wave of the COVID-19 pandemic (March 2021 to present)?

- Very unwilling
- Unwilling
- Neutral
- Willing
- Very willing

Q4b. How willing were you to provide *virtual* clinical care during the third wave of the COVID-19 pandemic (March 2021 to present)?

- Very unwilling
- Unwilling
- Neutral
- Willing
- Very willing

WILLINGNESS to work in other settings

**Q16. How comfortable are you with being redeployed to any of the following areas during the third wave of the COVID-19 pandemic (March 2021 to present)?**
(Very uncomfortable, somewhat uncomfortable, neutral, somewhat comfortable, very comfortable

|  | **Very uncomfortable** | **Somewhat uncomfortable** | **Neutral** | **Somewhat comfortable** | **Very comfortable** | **N/A – already work in this area** |
| --- | --- | --- | --- | --- | --- | --- |
| **Hospital inpatient care** |  |  |  |  |  |  |
| **Intensive Care Unit** |  |  |  |  |  |  |
| **COVID-19 Assessment/Testing Center** |  |  |  |  |  |  |
| **COVID-19 vaccination Center** |  |  |  |  |  |  |
| **Virtual COVID-19 Assessment/Follow-up** |  |  |  |  |  |  |
| **Palliative care** |  |  |  |  |  |  |
| **House calls** |  |  |  |  |  |  |
| **Emergency room shifts** |  |  |  |  |  |  |
| **Nursing home/ long-term care visits** |  |  |  |  |  |  |
| **Homeless shelters** |  |  |  |  |  |  |

**Q17. Which of the following do you consider barriers to being redeployed during the COVID-19 pandemic?** Select all that apply

- Lack of skills in area
- COVID-19 exposure risk and fears of contracting and transmitting virus
- Inadequate remuneration
- Time commitment
- Learning new environment (including safety procedures and logistics of clinical care)
- Finding coverage for current clinical practice
- Other (please specify): _______

Q18. Did you *start new work* in any of the following settings during the COVID-19 pandemic?

- Hospital inpatient care
- Intensive care unit
- COVID-19 assessment/testing centre
- COVID-19 vaccination centre
- Virtual COVID-19 assessments/follow-ups
- Palliative care
- House calls
- Emergency room shifts
- Nursing home/long-term care visits
- Homeless shelters

PREPAREDNESS

Q19. How supported do you feel in providing *in-person* clinical care during the third wave of the COVID-19 pandemic (March 2021 to present)?

- Very unsupported
- Somewhat unsupported
- Neutral
- Somewhat supported
- Very supported

Q19. How supported do you feel in providing *virtual* clinical care during the third wave of the COVID-19 pandemic (March 2021 to present)?

- Very unsupported
- Somewhat unsupported
- Neutral
- Somewhat supported
- Very supported

**Q20. Which of the following do you feel *needs improvement* in your clinical practice during the third wave of the COVID-19 pandemic (March 2021 to present)?**

- Implementing infection control practices for clinic space
- COVID19 testing
- Managing potential/confirmed COVID19 cases
- Obtaining Personal Protective Equipment (PPE)
- Obtaining disinfectant supplies
- Using PPE appropriately
- Implementing physical distancing in clinic
- Making use of an isolation room in clinic
- Managing staff/personal fear
- Providing/following leadership within the clinic
- Handling the non-specific symptoms and/or potential asymptomatic presentation of COVID-19
- Handling increased non-clinical responsibilities (e.g. care of dependents, etc.)

**Q22. Did you experience any of the following during the third wave of the COVID-19 pandemic (March 2021 to present)?**

- More doctor visits (virtual or in-person)
- Fewer doctor visits (virtual or in-person)
- More visits for depression/anxiety (virtual or in-person)
- Needed more reassurance from their physician.
- More appointment cancellations.
- More patients hesitant to go to an emergency room after being advised to go
- Patients presenting later in their illness with more acute concerns

**Q23. How safe do you feel travelling to and from your clinical environment during the third wave of the COVID-19 pandemic (March 2021 to present)?**

**Very unsafe, unsafe, Neutral, safe, Very safe, N/A**

**Q31. Have you ever received any formal training in the handling of an infectious disease outbreak in primary care prior to the COVID-19 pandemic?**

- Yes
- No

**Q32. What is your level of experience in handling or practicing during an infectious disease outbreak prior to the COVID-19 pandemic?**

- No experience
- Some experience
- Moderate experience
- Significant experience

Attitudes concerning COVID-19

**Q34. Please indicate your level of agreement for the following statements using the following scale:** Strongly Disagree, Disagree, Neutral, Agree, Strongly Agree, Not applicable

|  | **Strongly Disagree** | **Disagree** | **Neutral** | **Agree** | **Strongly Agree** | **N/A** |
| --- | --- | --- | --- | --- | --- | --- |
| I am frightened of dealing with COVID19 as a front-line doctor in the community. |  |  |  |  |  |  |
| I am worried about infecting my family due to my job. |  |  |  |  |  |  |
| My family is worried about being infected by me due to the nature of my job. |  |  |  |  |  |  |
| COVID19 is affecting my quality of life. |  |  |  |  |  |  |
| I am able to provide good clinical care during the COVID-19 pandemic |  |  |  |  |  |  |
| My work during the pandemic is valued. |  |  |  |  |  |  |
| I am satisfied with my ability to handle work responsibilities during the third wave of the COVID-19 pandemic |  |  |  |  |  |  |
| I am satisfied with my ability to handle my personal responsibilities during the third wave of the COVID-19 pandemic |  |  |  |  |  |  |
| I have a duty to provide clinical care during the COVID-19 pandemic |  |  |  |  |  |  |
| I am satisfied with the measures Public Health implemented to prevent COVID-19 from spreading in the community |  |  |  |  |  |  |

Personal Impact of COVID-19

**Q38. Have you received a dose of COVID-19 vaccine? (select one)**

- **No**
- **Yes, 1 dose**
- **Yes, 2 doses**

**Q39. Were you or your spouse pregnant during the third wave of the COVID-19 pandemic (March 2021 to present)?**

- Yes
- No

**Q40. Do you have any medical comorbidities that could put you at increased risk of COVID-19 related complications (excluding pregnancy)?**

- Yes
- No
- Unsure

**Q41. Does anybody in your household have any medical conditions/comorbidities that could put them at increased risk of COVID-19 related complications (excluding pregnancy)?**

- Yes
- No
- Unsure

**Q43. Did you lose clinical income due to the COVID-19?**

- No
- Yes
- Unsure

**Q46. Has COVID-19 affected your career in any of the following ways? Check all that apply.**

- Delayed starting clinical practice
- Took on less locum work than usual
- Took on more locum work than usual
- Have cut down clinical hours
- Have increased clinical hours
- Plan to retire earlier
- Plan to retire later
- Have come out of retirement
- Other: ______
- None of the above

**Q42. What precautions were taken to protect your family during the third wave of the COVID-19 pandemic (March 2021 to present)?** (Check all that apply).

- Stayed away from home
- Sent your family away
- Wore a mask in your home
- Washed your hands before entering your home
- Changed clothes before having contact with your family members
- Had a shower before having contact with your family members
- Used regular disinfectant to clean your home
- Other (please specify) _______

**Q47. Are you a parent to a child in your household?**

- Yes
- No

**Q48. If yes, how many children were living in your household during peak pandemic?** Please list: **____**

**Q49. What are the age groups of the children living in your household? (**Check all that apply)

- Infant (0-11 months)
- Toddler (1-2 years)
- Pre-school (3-4 years)
- School age (5-9 years)
- Pre-teen (10-12 years)
- Teenager (13-18 years)
- Young adult (18-25 years)
- Adult (26+ years)

**Q50. Was lack of childcare an issue in your household? (i.e. due to daycare/school closures)**

- Yes - Needed to modify childcare arrangements
- No - Childcare is not an issue in my household

**Q51. If yes, how did you manage?** (Check all that apply)

- Reduced clinic hours
- Closed practice due to childcare demands
- Partner had to reduce work hours
- Adjusted work schedule to work after-hours.
- Reduced non-clinical work activities due to childcare demands.
- Hired additional staff to care for children
- Enlisted friends/family to care for children
- Enrolled child/children in home daycare program
- Made use of Ontario’s emergency childcare for essential workers
- Other: ______

**Q52. Are you a caregiver to an elderly or disabled relative?**

- Yes
- No

**Q53. If yes, was the level of support you personally provided for your elderly or disabled relative affected during the COVID19 pandemic?**

- Yes – I provided less support
- Yes – I provided additional support
- No

**Q53. If yes, was the level of hired support for your elderly or disabled relative affected during the COVID19 pandemic?**

- Yes – hired supports were decreased
- Yes – hired supports were increased
- No
- N/A

Demographics and Practice Profile

**Q55. What is your age group?**

- Under 39
- 40-49
- 50-59
- 60 or over

**Q56. What best describes your current gender identity?**

- Male
- Female
- Something else (e.g. two-spirit, gender fluid, non-binary)
- Prefer not to answer

**Q58. How many years have you been in practice?**

- 0 years: First year of practice
- 1-5 years
- 6-10 years
- 11-20 years
- 20-30 years
- More than 30 years

**Q59. What best describes your primary practice community?**

- Urban >1,000,000 inhabitants
- Urban 100,00-999,999 inhabitants
- Urban 10,000-99,999 inhabitants
- Rural <10,000 inhabitants

**Q60. Is your primary practice located in the Greater Toronto Area (GTA*)?** *This response helps us assess the comparability of responses to previously administered surveys within the GTA.*

- Yes
- No

*GTA includes Burlington, Milton, Halton Hills, Oakville, Mississauga, Brampton, Caledon, Toronto, Vaughan, King, Newmarket, Aurora, Richmond Hill, East Gwillimbury, Whitchurch-Stouffville, Markham, Georgina, Uxbridge, Pickering, Ajax, Whitby, Oshawa, Scugog, Brock, Clarington

**Q62. Please list all practice settings in which you are involved.** *Please select all that apply.*

- Family medicine – general practice
- Family medicine - obstetrics
- Walk-in clinic
- Emergency Department
- Psychotherapy/counselling
- Sports Medicine
- Palliative Care
- Addictions Medicine
- Long-term Care
- Inpatient medicine
- Other: __________

**Q63. How are you mainly funded for your work as a family physician?** Please select one.

- Capitation
- Salary
- Fee-for-service
- Hourly/sessional fee
- Other: _________

**Q64. Are you working full time during the third wave of the COVID-19 pandemic (March 2021 to present)?**

- Yes
- No

**Q65. What allied health practitioners are available to your patients through your office/practice?**

- Nurses (e.g. RNs)
- Nurse Practitioners
- Social Workers
- Dietitians
- Pharmacists
- Occupational Therapist
- Other: ______

**Q66. How many physicians are included in your practice?** (If in solo practice, please indicate “1”) **______**

**Q67. Do you teach residents as a regular part of your primary care practice?**

- Yes
- No

**Q68. Is your practice affiliated with an Ontario Health Team (OHT)?**

- Yes. Please list: _____
- No

**Q71. Do you use an Electronic Medical Record (EMR) in your office for patient care**?

- Yes
- No

If you would like to enter our draw to win one of three $150 Visa gift cards, please enter your email below: _______

In order to understand the impact of your responses on various healthcare service outcomes, we ask that you provide your OHIP billing number to allow linkage of your survey information to routinely collected health information held at ICES. This is optional. Your billing number will not be shared to any outside parties and only aggregated data will be reported (no individual level data will be reported).

Entering your billing number for linkage will give you an additional entry to win one of three $150 Visa gift cards.

*Thank you for participating in our survey.*
