# Supplementary material for: Factors associated with plans for early retirement among Ontario family physicians during the COVID-19 pandemic: a cross-sectional study
Source: BMC Prim Care. 2024 Apr 18;25:118. doi: 10.1186/s12875-024-02374-9 (PMC11025226; doi:10.1186/s12875-024-02374-9)
Supplement: Supplementary file 2 — Supplementary Material 2 [file 12875_2024_2374_MOESM2_ESM.docx]

**Supporting information**

**S2 Table. Plans to retire earlier due to the COVID-19 pandemic among Ontario FPs during the first and third waves of the COVID-19 pandemic by survey item**

|  | 1st wave (April-June 2020) | | |  | 3rd wave (March-July 2021) | | |  | |  |  |
| --- | --- | --- | --- | --- | --- | --- | --- | --- | --- | --- | --- |
| Plan to retire earlier |  | No | Yes |  |  | No | Yes |  |  |  |  |
|  | Total (n=393) | 369 (93.9%) | 24 (6.1%) | p | Total (n=454) | 360 (79.3%) | 94 (20.7%) | p | | |  |
| **Willing to work in-person** | |  |  |  |  |  |  |  | | | |
| No | 101 (25.7%) | 89 (88.1%) | 12 (11.9%) | 0.01 | 53 (11.7%) | 40 (75.5%) | 13 (24.5%) | 0.58 | | |  |
| Yes | 292 (74.3%) | 280 (95.9%) | 12 (4.1%) |  | 401 (88.3%) | 320 (79.8%) | 81 (20.2%) |  | | |  |
| **Willing to work virtually** | |  |  |  |  |  |  |  | | | |
| No | 30 (7.6%) | 25-30 (>80.0%) | <5 (<20.0%) | 0.70 | 32 (7.1%) | 25 (78.1%) | 7 (21.9%) | 1.00 | | |  |
| Yes | 363 (92.4%) | 339-345 (93.4-95.0%) | 18-24 (5.0-6.6%) | | 421 (92.9%) | 335 (79.6%) | 86 (20.4%) |  | | | |
| **Supported to work in-person** | |  |  |  |  |  |  |  | | | |
| No | 237 (63.7%) | 218 (92.0%) | 19 (8.0%) | 0.16 | 209 (48.5%) | 148 (70.8%) | 61 (29.2%) | <0.01 | | |  |
| Yes | 135 (36.3%) | 130 (96.3%) | 5 (3.7%) |  | 222 (51.5%) | 189 (85.1%) | 33 (14.9%) |  | | |  |
| **Supported to work virtually** | |  |  |  |  |  |  |  | | | |
| No | 166 (44.6%) | 151 (91.0%) | 15 (9.0%) | 0.11 | 155 (36.1%) | 111 (71.6%) | 44 (28.4%) | 0.02 | | |  |
| Yes | 206 (55.4%) | 197 (95.6%) | 9 (4.4%) |  | 274 (63.9%) | 224 (81.8%) | 50 (18.2%) |  | | |  |
| **Needed improvement** | |  |  |  |  |  |  |  | | | |
| *Isolation room in clinic* | |  |  |  |  |  |  |  | | | |
| No | 293 (74.6%) | 275 (93.9%) | 18 (6.1%) | 1.00 | 363 (79.8%) | 294 (81.0%) | 69 (19.0%) | 0.11 | | |  |
| Yes | 100 (25.4%) | 94 (94.0%) | 6 (6.0%) |  | 92 (20.2%) | 67 (72.8%) | 25 (27.2%) |  | | |  |
| *Clinic leadership* |  |  |  |  |  |  |  |  | | |  |
| No | 322 (82.1%) | 304 (94.4%) | 18 (5.6%) | 0.52 | 392 (86.2%) | 316 (80.6%) | 76 (19.4%) | 0.13 | | |  |
| Yes | 70 (17.9%) | 65 (92.9%) | 5 (7.1%) |  | 63 (13.8%) | 45 (71.4%) | 18 (28.6%) |  | | |  |
| *Managing COVID testing* | |  |  |  |  |  |  |  | | | |
| No | 255 (64.9%) | 242 (94.9%) | 13 (5.1%) | 0.36 | 366 (80.4%) | 291 (79.5%) | 75 (20.5%) | 0.97 | | |  |
| Yes | 138 (35.1%) | 127 (92.0%) | 11 (8.0%) |  | 89 (19.6%) | 70 (78.7%) | 19 (21.3%) |  | | |  |
| *Managing potential COVID cases* | |  |  |  |  |  |  |  | | | |
| No | 242 (61.6%) | 229 (94.6%) | 13 (5.4%) | 0.58 | 342 (75.2%) | 273 (79.8%) | 69 (20.2%) | 0.76 | | |  |
| Yes | 151 (38.4%) | 140 (92.7%) | 11 (7.3%) |  | 113 (24.8%) | 88 (77.9%) | 25 (22.1%) |  | | |  |
| *Using PPE appropriately* | |  |  |  |  |  |  |  | | | |
| No | 303 (77.1%) | 284 (93.7%) | 19 (6.3%) | 1.00 | 418 (91.9%) | 336 (80.4%) | 82 (19.6%) | 0.10 | | |  |
| Yes | 90 (22.9%) | 85 (94.4%) | 5 (5.6%) |  | 37 (8.1%) | 25 (67.6%) | 12 (32.4%) |  | | |  |
| *Managing staff/personal fear* | |  |  |  |  |  |  |  | | | |
| No | 204 (51.9%) | 194 (95.1%) | 10 (4.9%) | 0.41 | 333 (73.2%) | 274 (82.3%) | 59 (17.7%) | 0.02 | | |  |
| Yes | 189 (48.1%) | 175 (92.6%) | 14 (7.4%) |  | 122 (26.8%) | 87 (71.3%) | 35 (28.7%) |  | | |  |
| *Obtaining PPE* |  |  |  |  |  |  |  |  | | |  |
| No | 139 (35.4%) | 130 (93.5%) | 9 (6.5%) | 1.00 | 360 (79.1%) | 295 (81.9%) | 65 (18.1%) | 0.01 | | |  |
| Yes | 254 (64.6%) | 239 (94.1%) | 15 (5.9%) |  | 95 (20.9%) | 66 (69.5%) | 29 (30.5%) |  | | |  |
| *Physical distancing in clinic* | |  |  |  |  |  |  |  | | | |
| No | 248 (63.1%) | 235 (94.8%) | 13 (5.2%) | 0.47 | 357 (78.5%) | 285 (79.8%) | 72 (20.2%) | 0.72 | | |  |
| Yes | 145 (36.9%) | 134 (92.4%) | 11 (7.6%) |  | 98 (21.5%) | 76 (77.6%) | 22 (22.4%) |  | | |  |
| *Infection control practices in clinic* | |  |  |  |  |  |  |  | | | |
| No | 199 (50.6%) | 187 (94.0%) | 12 (6.0%) | 1.00 | 386 (84.8%) | 313 (81.1%) | 73 (18.9%) | 0.04 | | |  |
| Yes | 194 (49.4%) | 182 (93.8%) | 12 (6.2%) |  | 69 (15.2%) | 48 (69.6%) | 21 (30.4%) |  | | |  |
| *Handling increased non-clinical responsibilities* | | |  |  |  |  |  |  | |  |  |
| No | 243 (61.8%) | 236 (97.1%) | 7 (2.9%) | <0.01 | 285 (62.6%) | 243 (85.3%) | 42 (14.7%) | <0.01 | | |  |
| Yes | 150 (38.2%) | 133 (88.7%) | 17 (11.3%) |  | 170 (37.4%) | 118 (69.4%) | 52 (30.6%) |  | | |  |
| **Felt safe travelling to clinic** | |  |  |  |  |  |  |  | | | |
| No | 61 (16.5%) | 51 (83.6%) | 10 (16.4%) | <0.01 | 50 (11.7%) | 35 (70.0%) | 15 (30.0%) | 0.19 | | |  |
| Yes | 309 (83.5%) | 295 (95.5%) | 14 (4.5%) |  | 377 (88.3%) | 299 (79.3%) | 78 (20.7%) |  | | |  |
| **Training in infectious disease outbreaks** | |  |  |  |  |  |  |  | | | |
| No | 323 (86.8%) | 304 (94.1%) | 19 (5.9%) | 0.40 | 368 (85.4%) | 285 (77.4%) | 83 (22.6%) | 0.46 | | |  |
| Yes | 49 (13.2%) | 44 (89.8%) | 5 (10.2%) |  | 63 (14.6%) | 52 (82.5%) | 11 (17.5%) |  | | |  |
| **Experience with infectious disease outbreaks** | | |  |  |  |  |  |  | |  |  |
| No | 219 (58.9%) | 208 (95.0%) | 11 (5.0%) | 0.26 | 181 (42.0%) | 150 (82.9%) | 31 (17.1%) | 0.06 | | |  |
| Yes | 153 (41.1%) | 140 (91.5%) | 13 (8.5%) |  | 250 (58.0%) | 187 (74.8%) | 63 (25.2%) |  | | |  |
| **Frightened of Dealing with COVID-19** | |  |  |  |  |  |  |  | | | |
| No | 144 (39.2%) | 139 (96.5%) | 5 (3.5%) | 0.12 | 297 (69.6%) | 238 (80.1%) | 59 (19.9%) | 0.14 | | |  |
| Yes | 223 (60.8%) | 205 (91.9%) | 18 (8.1%) |  | 130 (30.4%) | 95 (73.1%) | 35 (26.9%) |  | | |  |
| **Worried about infecting family** | |  |  |  |  |  |  |  | | | |
| No | 51 (13.9%) | 46-51 (>90.2%) | <5 (9.8%) | 0.55 | 155 (36.6%) | 124 (80.0%) | 31 (20.0%) | 0.60 | | |  |
| Yes | 315 (86.1%) | 291-297 (92.4-94.3%) | 18-24 (8.1-10.8%) | | 269 (63.4%) | 208 (77.3%) | 61 (22.7%) |  | | | |
| **Family worried about getting infected** | |  |  |  |  |  |  |  | | | |
| No | 110 (30.1%) | 105 (95.5%) | 5 (4.5%) | 0.43 | 208 (49.2%) | 164 (78.8%) | 44 (21.2%) | 0.86 | | |  |
| Yes | 256 (69.9%) | 237 (92.6%) | 19 (7.4%) |  | 215 (50.8%) | 167 (77.7%) | 48 (22.3%) |  | | |  |
| **Provided good clinical care** | |  |  |  |  |  |  |  | | | |
| No | 107 (29.0%) | 101 (94.4%) | 6 (5.6%) | 0.83 | 141 (33.0%) | 102 (72.3%) | 39 (27.7%) | 0.06 | | |  |
| Yes | 262 (71.0%) | 244 (93.1%) | 18 (6.9%) |  | 286 (67.0%) | 231 (80.8%) | 55 (19.2%) |  | | |  |
| **Work during the pandemic was valued** | |  |  |  |  |  |  |  | | | |
| No | 110 (29.8%) | 104 (94.5%) | 6 (5.5%) | 0.76 | 146 (34.3%) | 106 (72.6%) | 40 (27.4%) | 0.07 | | |  |
| Yes | 259 (70.2%) | 241 (93.1%) | 18 (6.9%) |  | 280 (65.7%) | 226 (80.7%) | 54 (19.3%) |  | | |  |
| **Satisfied with ability to handle work responsibilities during the pandemic** | | | | |  |  |  |  | |  |  |
| No | 120 (32.6%) | 107 (89.2%) | 13 (10.8%) | 0.04 | 159 (37.5%) | 106 (66.7%) | 53 (33.3%) | <0.01 | | |  |
| Yes | 248 (67.4%) | 237 (95.6%) | 11 (4.4%) |  | 265 (62.5%) | 225 (84.9%) | 40 (15.1%) |  | | |  |
| **Satisfied with ability to handle personal responsibilities during the pandemic** | | | | |  |  |  |  | |  |  |
| No | 145 (39.3%) | 137 (94.5%) | 8 (5.5%) | 0.69 | 212 (49.9%) | 160 (75.5%) | 52 (24.5%) | 0.28 | | |  |
| Yes | 224 (60.7%) | 208 (92.9%) | 16 (7.1%) |  | 213 (50.1%) | 171 (80.3%) | 42 (19.7%) |  | | |  |
| **Duty to provide care during the pandemic** | |  |  |  |  |  |  |  | | | |
| No | 17 (4.6%) | 12-17 (>70.6%) | <5 (<29.4%) | 0.09 | 18 (4.2%) | 10 (55.6%) | 8 (44.4%) | 0.04 | | |  |
| Yes | 350 (95.4%) | 326-331 (93.1-94.6%) | 19-24 (5.4-6.9%) | | 406 (95.8%) | 321 (79.1%) | 85 (20.9%) |  | | | |
| **Satisfied with public health measures to prevent community spread** | | | |  |  |  |  |  | |  |  |
| No | 225 (61.0%) | 207 (92.0%) | 18 (8.0%) | 0.21 | 209 (48.9%) | 158 (75.6%) | 51 (24.4%) | 0.24 | | |  |
| Yes | 144 (39.0%) | 138 (95.8%) | 6 (4.2%) |  | 218 (51.1%) | 176 (80.7%) | 42 (19.3%) |  | | |  |
| **Lost Income** |  |  |  |  |  |  |  |  | | |  |
| No | 117 (31.8%) | 107-112 (91.5-95.7%) | 5-10 (4.3-8.5%) | 0.66 | 154 (36.3%) | 124 (80.5%) | 30 (19.5%) | 0.24 | | |  |
| Unsure | 48 (13.0%) | 43-48 (>89.6%) | <5 (<10.4%) |  | 52 (12.3%) | 36 (69.2%) | 16 (30.8%) |  | | |  |
| Yes | 203 (55.2%) | 189 (93.1%) | 14 (6.9%) |  | 218 (51.4%) | 170 (78.0%) | 48 (22.0%) |  | | |  |
| **Decreased hours** |  |  |  |  |  |  |  |  | | |  |
| No | 303 (77.1%) | 289 (95.4%) | 14 (4.6%) | 0.04 | 355 (78.0%) | 286 (80.6%) | 69 (19.4%) | 0.28 | | |  |
| Yes | 90 (22.9%) | 80 (88.9%) | 10 (11.1%) |  | 100 (22.0%) | 75 (75.0%) | 25 (25.0%) |  | | |  |
| **Increased hours** |  |  |  |  |  |  |  |  | | |  |
| No | 297 (75.6%) | 273-278 (91.9-93.6%) | 19-24 (6.4-8.1%) | 0.47 | 291 (74.4%) | 222 (76.3%) | 69 (23.7%) | 1.00 | | |  |
| Yes | 96 (24.4%) | 91-96 (>94.8%) | <5 (5.2%) |  | 100 (25.6%) | 75 (75.0%) | 25 (25.0%) |  | | |  |
| **Pregnancy (You or spouse)** | |  |  |  |  |  |  |  | | | |
| No | 338 (91.4%) | 314-319 (92.9-94.4%) | 19-24 (5.6-7.1%) | 1.00 | 406 (95.8%) | 312-317 (76.8-78.1%) | 89-94 (21.9-23.2%) | 0.14 | | |  |
| Yes | 32 (8.6%) | 27-32 (>84.4%) | <5 (<15.6%) |  | 18 (4.2%) | 13-18 (>72.2%) | <5 (<27.8%) |  | | |  |
| **Personal medical conditions** | |  |  |  |  |  |  |  | | | |
| No | 298 (81.0%) | 282 (94.6%) | 16 (5.4%) | 0.13 | 315 (74.3%) | 253 (80.3%) | 62 (19.7%) | 0.09 | | |  |
| Yes | 65-70 (17.7-19.0%) | 57-62 (81.4-95.4%) | 3-8 (4.3-22.9%) | | 102 (24.1%) | 70-75 (68.6-73.5%) | 27-32 (26.5-31.4%) | | |  |  |
| Unsure | <5 (<1.4%) | <5 | <5 |  | 7 (1.7%) | 2-7 (>28.6%) | <5 (<71.4%) |  | | |  |
| **Family member with medical conditions** | |  |  |  |  |  |  |  | | | |
| No | 271 (73.2%) | 256 (94.5%) | 15 (5.5%) | 0.28 | 253 (59.7%) | 200 (79.1%) | 53 (20.9%) | 0.60 | | |  |
| Yes | 94-99 (45.4-26.8%) | 85-90 (85.9-95.7%) | 4-9 (4.0-9.6%) | | 165 (38.9%) | 126 (76.4%) | 39 (23.6%) |  | | | |
| Unsure | <5 (<1.4%) | <5 | <5 |  | 6 (1.4%) | 1-6 (>16.7%) | <5 (<83.3%) |  | | |  |
| **Parent** |  |  |  |  |  |  |  |  | | |  |
| No | 146 (39.5%) | 136 (93.2%) | 10 (6.8%) | 0.99 | 174 (41.1%) | 131 (75.3%) | 43 (24.7%) | 0.36 | | |  |
| Yes | 224 (60.5%) | 210 (93.8%) | 14 (6.3%) |  | 249 (58.9%) | 198 (79.5%) | 51 (20.5%) |  | | |  |
| **Care for elderly relative** | |  |  |  |  |  |  |  | | | |
| No | 331 (89.7%) | 307-312 (92.7-94.3%) | 19-24 (5.7-7.3%) | 0.29 | 325 (77.0%) | 267 (82.2%) | 58 (17.8%) | <0.01 | | |  |
| Yes | 38 (10.3%) | 33-38 (>86.8%) | <5 (<13.2%) |  | 97 (23.0%) | 62 (63.9%) | 35 (36.1%) |  | | |  |
| **Age** |  |  |  |  |  |  |  |  | | |  |
| Under 39 | 188 (51.2%) | 181 (96.3%) | 7 (3.7%) | 0.08 | 88 (21.0%) | 80 (90.9%) | 8 (9.1%) | <0.01 | | |  |
| 40-49 | 73 (19.9%) | 67 (91.8%) | 6 (8.2%) |  | 104 (24.8%) | 91 (87.5%) | 13 (12.5%) |  | | |  |
| 50-59 | 63 (17.2%) | 58 (92.1%) | 5 (7.9%) |  | 116 (27.6%) | 77 (66.4%) | 39 (33.6%) |  | | |  |
| 60 or over | 43 (11.7%) | 37 (86.0%) | 6 (14.0%) |  | 112 (26.7%) | 79 (70.5%) | 33 (29.5%) |  | | |  |
| **Gender** |  |  |  |  |  |  |  |  | | |  |
| Female | 244 (66.5%) | 231 (94.7%) | 13 (5.3%) | 0.29 | 276 (65.7%) | 215 (77.9%) | 61 (22.1%) | 1.00 | | |  |
| Male | 118-123 (32.2-33.5%) | 107-112 (87.0-94.9%) | 6-11 (4.9-9.3%) | | 144 (34.3%) | 112 (77.8%) | 32 (22.2%) |  | | | |
| Other/non-binary | <5 (<1.4%) | <5 | <5 |  |  |  |  |  | | |  |
| **Community size (population)** | |  |  |  |  |  |  |  | | | |
| Rural (<10k) | 61 (16.9%) | 55-60 (90.2-98.4%) | 1-6 (1.6-9.8%) | 0.76 | 33 (7.9%) | 24 (72.7%) | 9 (27.3%) | 0.61 | | |  |
| Urban (10-100k) | 62 (17.2%) | 57-62 (>91.9%) | <5 (<8.1%) |  | 78 (18.6%) | 64 (82.1%) | 14 (17.9%) |  | | |  |
| Urban (100k-1M) | 80 (22.2%) | 74 (92.5%) | 6 (7.5%) |  | 148 (35.2%) | 112 (75.7%) | 36 (24.3%) |  | | |  |
| Urban (>1M) | 157 (43.6%) | 145 (92.4%) | 12 (7.6%) |  | 161 (38.3%) | 127 (78.9%) | 34 (21.1%) |  | | |  |
| **GTA** |  |  |  |  |  |  |  |  | | |  |
| Yes | 181 (49.3%) | 166 (91.7%) | 15 (8.3%) | 0.26 | 210 (50.0%) | 165 (78.6%) | 45 (21.4%) | 0.81 | | |  |
| No | 186 (50.7%) | 177 (95.2%) | 9 (4.8%) |  | 210 (50.0%) | 162 (77.1%) | 48 (22.9%) |  | | |  |
| **Specialized/focused practice** | |  |  |  |  |  |  |  | | | |
| No | 178 (45.3%) | 165 (92.7%) | 13 (7.3%) | 0.49 | 242 (53.2%) | 190 (78.5%) | 52 (21.5%) | 0.73 | | |  |
| Yes | 215 (54.7%) | 204 (94.9%) | 11 (5.1%) |  | 213 (46.8%) | 171 (80.3%) | 42 (19.7%) |  | | |  |
| **Any allied health available through office/practice** | | |  |  |  |  |  |  | |  |  |
| No | 87 (22.1%) | 80 (92.0%) | 7 (8.0%) | 0.55 | 127 (27.9%) | 99 (78.0%) | 28 (22.0%) | 0.74 | | |  |
| Yes | 306 (77.9%) | 289 (94.4%) | 17 (5.6%) |  | 328 (72.1%) | 262 (79.9%) | 66 (20.1%) |  | | |  |
| **Number of Physicians in Practice** | |  |  |  |  |  |  |  | | | |
| 1 | 39 (10.9%) | 31 (79.5%) | 8 (20.5%) | 0.01 | 83 (20.1%) | 62 (74.7%) | 21 (25.3%) | 0.07 | | |  |
| 2 | 22 (6.1%) | 17-22 (>77.3%) | <5 (<22.7%) |  | 50 (12.1%) | 32 (64.0%) | 18 (36.0%) |  | | |  |
| 3-4 | 66 (18.4%) | 61-66 (>92.4%) | <5 (<7.6%) |  | 95 (23.1%) | 76 (80.0%) | 19 (20.0%) |  | | |  |
| 5-9 | 151 (42.2%) | 143 (94.7%) | 8 (5.3%) |  | 108 (26.2%) | 91 (84.3%) | 17 (15.7%) |  | | |  |
| 10+ | 80 (22.3%) | 75 (93.8%) | 5 (6.3%) |  | 76 (18.4%) | 59 (77.6%) | 17 (22.4%) |  | | |  |
| **Payment Model** |  |  |  |  |  |  |  |  | | |  |
| Fee-for-service | 70 (19.1%) | 65-70 (>92.9%) | <5 (<7.1%) | 0.44 | 136 (32.5%) | 101 (74.3%) | 35 (25.7%) | 0.39 | | |  |
| Capitation | 231 (63.1%) | 215 (93.1%) | 16 (6.9%) |  | 231 (55.1%) | 182 (78.8%) | 49 (21.2%) |  | | |  |
| Hourly/Sessional fee | 10 (2.7%) | 5-10 (>50.0%) | <5 (<50%) |  | 13 (3.1%) | 8-13 (>61.5%) | <5 (<38.5%) |  | | |  |
| Other | 27 (7.4%) | 22-25 (>81.5%) | <5 (<18.5%) |  | 17 (4.1%) | 12-17 (>70.6%) | <5 (<29.4%) |  | | |  |
| Salary | 28 (7.7%) | 23-28 (>82.1%) | <5 (<17.9%) |  | 22 (5.3%) | 16 (72.7%) | 6 (27.3%) |  | | |  |
| **Working Fulltime pre-pandemic** | |  |  |  |  |  |  |  | | | |
| Yes | 308 (83.9%) | 291 (94.5%) | 17 (5.5%) | 0.13 | 350 (83.5%) | 271 (77.4%) | 79 (22.6%) | 0.54 | | |  |
| No | 59 (16.1%) | 52 (88.1%) | 7 (11.9%) |  | 69 (16.5%) | 55 (79.7%) | 14 (20.3%) |  | | |  |
| **Works with Residents** | |  |  |  |  |  |  |  | | | |
| No | 217 (59.1%) | 202 (93.1%) | 15 (6.9%) | 0.89 | 290 (69.2%) | 221 (76.2%) | 69 (23.8%) | 0.29 | | |  |
| Yes | 150 (40.9%) | 141 (94.0%) | 9 (6.0%) |  | 129 (30.8%) | 105 (81.4%) | 24 (18.6%) |  | | |  |
| **Works in an OHT** |  |  |  |  |  |  |  |  | | |  |
| No | 230 (63.0%) | 212 (92.2%) | 18 (7.8%) | 0.30 | 285 (68.2%) | 220 (77.2%) | 65 (22.8%) | 0.78 | | |  |
| Yes | 135 (37.0%) | 129 (95.6%) | 6 (4.4%) |  | 133 (31.8%) | 105 (78.9%) | 28 (21.1%) |  | | |  |
| **Uses EMR** |  |  |  |  |  |  |  |  | | |  |
| Yes | 350 (97.8%) | 338 (96.6%) | 12 (3.4%) | 0.01 | 386 (91.9%) | 305 (79.0%) | 81 (21.0%) | 0.09 | | |  |
| No | 8 (2.2%) | 3-8 (>37.5%) | <5 (<62.5%) |  | 34 (8.1%) | 22 (64.7%) | 12 (35.3%) |  | | |  |

**S3 Table. Plans to retire earlier due to the COVID-19 pandemic among Ontario FPs during the first and third waves of the COVID-19 pandemic by non-dichotomized survey item. Only survey items that were dichotomized are included**

|  | 1st wave (April-June 2020) | | |  | 3rd wave (March-July 2021) | | |  |
| --- | --- | --- | --- | --- | --- | --- | --- | --- |
| Plan to retire earlier | | No | Yes |  |  | No | Yes |  |
|  | Total (n=393) | 369 (93.9%) | 24 (6.1%) | p | Total (n=454) | 360 (79.3%) | 94 (20.7%) | p |
| **Willing to work in-person** | |  |  |  |  |  |  |  |
| Neutral | 51 (12.98%) | 47 (92.16%) | 4 (7.84%) | <0.001 | 26 (5.73%) | 17 (65.38%) | 9 (34.62%) | 0.223 |
| Very unwilling | 10 (2.54%) | 8 (80.00%) | 2 (20.00%) |  | 9 (1.98%) | 9 (100.00%) | 0 (0.00%) |  |
| Unwilling | 40 (10.18%) | 34 (85.00%) | 6 (15.00%) |  | 18 (3.96%) | 14 (77.78%) | 4 (22.22%) |  |
| Willing | 181 (46.06%) | 169 (93.37%) | 12 (6.63%) |  | 171 (37.67%) | 139 (81.29%) | 32 (18.71%) |  |
| Very willing | 111 (28.24%) | 111 (100.00%) | 0 (0.00%) |  | 230 (50.66%) | 181 (78.70%) | 49 (21.30%) |  |
| **Willing to work virtually** | |  |  |  |  |  |  |  |
| Neutral | 13 (3.31%) | 12 (92.31%) | 1 (7.69%) | 0.150 | 13 (2.87%) | 10 (76.92%) | 3 (23.08%) | 0.459 |
| Very unwilling | 16 (4.07%) | 15 (93.75%) | 1 (6.25%) |  | 16 (3.53%) | 13 (81.25%) | 3 (18.75%) |  |
| Unwilling | 1 (0.25%) | 1 (100.00%) | 0 (0.00%) |  | 3 (0.66%) | 2 (66.67%) | 1 (33.33%) |  |
| Willing | 65 (16.54%) | 57 (87.69%) | 8 (12.31%) |  | 90 (19.87%) | 77 (85.56%) | 13 (14.44%) |  |
| Very willing | 298 (75.83%) | 284 (95.30%) | 14 (4.70%) |  | 331 (73.07%) | 258 (77.95%) | 73 (22.05%) |  |
| **Supported to work in-person** | | |  |  |  |  |  |  |
| Neutral | 55 (14.78%) | 52 (94.55%) | 3 (5.45%) | 0.253 | 100 (23.20%) | 79 (79.00%) | 21 (21.00%) | <0.001 |
| Very unsupported | 75 (20.16%) | 66 (88.00%) | 9 (12.00%) |  | 41 (9.51%) | 21 (51.22%) | 20 (48.78%) |  |
| Somewhat unsupported | 107 (28.76%) | 100 (93.46%) | 7 (6.54%) |  | 68 (15.78%) | 48 (70.59%) | 20 (29.41%) |  |
| Somewhat supported | 95 (25.54%) | 92 (96.84%) | 3 (3.16%) |  | 138 (32.02%) | 115 (83.33%) | 23 (16.67%) |  |
| Very supported | 40 (10.75%) | 38 (95.00%) | 2 (5.00%) |  | 84 (19.49%) | 74 (88.10%) | 10 (11.90%) |  |
| **Supported to work virtually** | |  |  |  |  |  |  |  |
| Neutral | 72 (19.35%) | 66 (91.67%) | 6 (8.33%) | 0.405 | 87 (20.28%) | 69 (79.31%) | 18 (20.69%) | 0.001 |
| Very unsupported | 29 (7.80%) | 26 (89.66%) | 3 (10.34%) |  | 16 (3.73%) | 8 (50.00%) | 8 (50.00%) |  |
| Somewhat unsupported | 65 (17.47%) | 59 (90.77%) | 6 (9.23%) |  | 52 (12.12%) | 34 (65.38%) | 18 (34.62%) |  |
| Somewhat supported | 131 (35.22%) | 125 (95.42%) | 6 (4.58%) |  | 155 (36.13%) | 120 (77.42%) | 35 (22.58%) |  |
| Very supported | 75 (20.16%) | 72 (96.00%) | 3 (4.00%) |  | 119 (27.74%) | 104 (87.39%) | 15 (12.61%) |  |
| **Felt safe travelling to clinic** | |  |  |  |  |  |  |  |
| Neutral | 37 (10.00%) | 34 (91.89%) | 3 (8.11%) | 0.001 | 38 (8.90%) | 26 (68.42%) | 12 (31.58%) | 0.414 |
| Very unsafe | 7 (1.89%) | 5 (71.43%) | 2 (28.57%) |  | 2 (0.47%) | 2 (100.00%) | 0 (0.00%) |  |
| Unsafe | 17 (4.59%) | 12 (70.59%) | 5 (29.41%) |  | 10 (2.34%) | 7 (70.00%) | 3 (30.00%) |  |
| Safe | 108 (29.19%) | 105 (97.22%) | 3 (2.78%) |  | 99 (23.19%) | 81 (81.82%) | 18 (18.18%) |  |
| Very safe | 201 (54.32%) | 190 (94.53%) | 11 (5.47%) |  | 278 (65.11%) | 218 (78.42%) | 60 (21.58%) |  |
| **Experience with infectious disease outbreaks** | | | |  |  |  |  |  |
| No experience | 219 (58.87%) | 208 (94.98%) | 11 (5.02%) | 0.148 | 181 (42.00%) | 150 (82.87%) | 31 (17.13%) | 0.077 |
| Some experience | 107 (28.76%) | 95 (88.79%) | 12 (11.21%) |  | 180 (41.76%) | 130 (72.22%) | 50 (27.78%) |  |
| Moderate experience | 35 (9.41%) | 34 (97.14%) | 1 (2.86%) |  | 53 (12.30%) | 44 (83.02%) | 9 (16.98%) |  |
| Significant experience | 11 (2.96%) | 11 (100.00%) | 0 (0.00%) |  | 17 (3.94%) | 13 (76.47%) | 4 (23.53%) |  |
| **Frightened of Dealing with COVID-19** | | |  |  |  |  |  |  |
| Neutral | 72 (19.62%) | 71 (98.61%) | 1 (1.39%) | 0.195 | 88 (20.61%) | 66 (75.00%) | 22 (25.00%) | 0.146 |
| Strongly Disagree | 14 (3.81%) | 13 (92.86%) | 1 (7.14%) |  | 78 (18.27%) | 61 (78.21%) | 17 (21.79%) |  |
| Disagree | 58 (15.80%) | 55 (94.83%) | 3 (5.17%) |  | 131 (30.68%) | 111 (84.73%) | 20 (15.27%) |  |
| Agree | 167 (45.50%) | 155 (92.81%) | 12 (7.19%) |  | 97 (22.72%) | 73 (75.26%) | 24 (24.74%) |  |
| Strongly Agree | 56 (15.26%) | 50 (89.29%) | 6 (10.71%) |  | 33 (7.73%) | 22 (66.67%) | 11 (33.33%) |  |
| **Worried about infecting family** | | |  |  |  |  |  |  |
| Neutral | 21 (5.74%) | 21 (100.00%) | 0 (0.00%) | 0.519 | 49 (11.56%) | 43 (87.76%) | 6 (12.24%) | 0.341 |
| Strongly Disagree | 8 (2.19%) | 7 (87.50%) | 1 (12.50%) |  | 36 (8.49%) | 25 (69.44%) | 11 (30.56%) |  |
| Disagree | 22 (6.01%) | 21 (95.45%) | 1 (4.55%) |  | 70 (16.51%) | 56 (80.00%) | 14 (20.00%) |  |
| Agree | 171 (46.72%) | 161 (94.15%) | 10 (5.85%) |  | 167 (39.39%) | 129 (77.25%) | 38 (22.75%) |  |
| Strongly Agree | 144 (39.34%) | 132 (91.67%) | 12 (8.33%) |  | 102 (24.06%) | 79 (77.45%) | 23 (22.55%) |  |
| **Family worried about getting infected** | | |  |  |  |  |  |  |
| Neutral | 53 (14.48%) | 49 (92.45%) | 4 (7.55%) | 0.707 | 76 (17.97%) | 58 (76.32%) | 18 (23.68%) | 0.255 |
| Strongly Disagree | 10 (2.73%) | 10 (100.00%) | 0 (0.00%) |  | 37 (8.75%) | 28 (75.68%) | 9 (24.32%) |  |
| Disagree | 47 (12.84%) | 46 (97.87%) | 1 (2.13%) |  | 95 (22.46%) | 78 (82.11%) | 17 (17.89%) |  |
| Agree | 153 (41.80%) | 141 (92.16%) | 12 (7.84%) |  | 130 (30.73%) | 107 (82.31%) | 23 (17.69%) |  |
| Strongly Agree | 103 (28.14%) | 96 (93.20%) | 7 (6.80%) |  | 85 (20.09%) | 60 (70.59%) | 25 (29.41%) |  |
| **Provided good clinical care** | |  |  |  |  |  |  |  |
| Neutral | 63 (17.07%) | 61 (96.83%) | 2 (3.17%) | 0.030 | 84 (19.67%) | 61 (72.62%) | 23 (27.38%) | 0.204 |
| Strongly Disagree | 4 (1.08%) | 2 (50.00%) | 2 (50.00%) |  | 8 (1.87%) | 6 (75.00%) | 2 (25.00%) |  |
| Disagree | 40 (10.84%) | 38 (95.00%) | 2 (5.00%) |  | 49 (11.48%) | 35 (71.43%) | 14 (28.57%) |  |
| Agree | 183 (49.59%) | 173 (94.54%) | 10 (5.46%) |  | 198 (46.37%) | 156 (78.79%) | 42 (21.21%) |  |
| Strongly Agree | 79 (21.41%) | 71 (89.87%) | 8 (10.13%) |  | 88 (20.61%) | 75 (85.23%) | 13 (14.77%) |  |
| **Work during the pandemic was valued** | | |  |  |  |  |  |  |
| Neutral | 67 (18.16%) | 64 (95.52%) | 3 (4.48%) | 0.505 | 81 (19.01%) | 60 (74.07%) | 21 (25.93%) | 0.060 |
| Strongly Disagree | 12 (3.25%) | 10 (83.33%) | 2 (16.67%) |  | 23 (5.40%) | 18 (78.26%) | 5 (21.74%) |  |
| Disagree | 31 (8.40%) | 30 (96.77%) | 1 (3.23%) |  | 42 (9.86%) | 28 (66.67%) | 14 (33.33%) |  |
| Agree | 164 (44.44%) | 153 (93.29%) | 11 (6.71%) |  | 178 (41.78%) | 137 (76.97%) | 41 (23.03%) |  |
| Strongly Agree | 95 (25.75%) | 88 (92.63%) | 7 (7.37%) |  | 102 (23.94%) | 89 (87.25%) | 13 (12.75%) |  |
| **Satisfied with ability to handle work responsibilities during the pandemic** | | | | | |  |  |  |
| Neutral | 67 (18.21%) | 62 (92.54%) | 5 (7.46%) | 0.063 | 76 (17.92%) | 56 (73.68%) | 20 (26.32%) | <0.001 |
| Strongly Disagree | 11 (2.99%) | 9 (81.82%) | 2 (18.18%) |  | 15 (3.54%) | 6 (40.00%) | 9 (60.00%) |  |
| Disagree | 42 (11.41%) | 36 (85.71%) | 6 (14.29%) |  | 68 (16.04%) | 44 (64.71%) | 24 (35.29%) |  |
| Agree | 166 (45.11%) | 159 (95.78%) | 7 (4.22%) |  | 187 (44.10%) | 159 (85.03%) | 28 (14.97%) |  |
| Strongly Agree | 82 (22.28%) | 78 (95.12%) | 4 (4.88%) |  | 78 (18.40%) | 66 (84.62%) | 12 (15.38%) |  |
| **Satisfied with ability to handle personal responsibilities during the pandemic** | | | | | |  |  |  |
| Neutral | 74 (20.05%) | 69 (93.24%) | 5 (6.76%) | 0.911 | 98 (23.06%) | 81 (82.65%) | 17 (17.35%) | 0.024 |
| Strongly Disagree | 19 (5.15%) | 19 (100.00%) | 0 (0.00%) |  | 33 (7.76%) | 19 (57.58%) | 14 (42.42%) |  |
| Disagree | 52 (14.09%) | 49 (94.23%) | 3 (5.77%) |  | 81 (19.06%) | 60 (74.07%) | 21 (25.93%) |  |
| Agree | 162 (43.90%) | 151 (93.21%) | 11 (6.79%) |  | 151 (35.53%) | 119 (78.81%) | 32 (21.19%) |  |
| Strongly Agree | 62 (16.80%) | 57 (91.94%) | 5 (8.06%) |  | 62 (14.59%) | 52 (83.87%) | 10 (16.13%) |  |
| **Duty to provide care during the pandemic** | | |  |  |  |  |  |  |
| Neutral | 12 (3.27%) | 10 (83.33%) | 2 (16.67%) | 0.188 | 15 (3.54%) | 10 (66.67%) | 5 (33.33%) | 0.017 |
| Strongly Disagree | 1 (0.27%) | 1 (100.00%) | 0 (0.00%) |  | 3 (0.71%) | 0 (0.00%) | 3 (100.00%) |  |
| Disagree | 4 (1.09%) | 3 (75.00%) | 1 (25.00%) |  | 0 (0.00%) | 0 (0.00%) | 0 (0.00%) |  |
| Agree | 112 (30.52%) | 105 (93.75%) | 7 (6.25%) |  | 148 (34.91%) | 117 (79.05%) | 31 (20.95%) |  |
| Strongly Agree | 238 (64.85%) | 224 (94.12%) | 14 (5.88%) |  | 258 (60.85%) | 204 (79.07%) | 54 (20.93%) |  |
| **Satisfied with public health measures to prevent community spread** | | | | | |  |  |  |
| Neutral | 88 (23.85%) | 83 (94.32%) | 5 (5.68%) | 0.379 | 94 (22.01%) | 77 (81.91%) | 17 (18.09%) | 0.004 |
| Strongly Disagree | 49 (13.28%) | 44 (89.80%) | 5 (10.20%) |  | 46 (10.77%) | 26 (56.52%) | 20 (43.48%) |  |
| Disagree | 88 (23.85%) | 80 (90.91%) | 8 (9.09%) |  | 69 (16.16%) | 55 (79.71%) | 14 (20.29%) |  |
| Agree | 113 (30.62%) | 109 (96.46%) | 4 (3.54%) |  | 153 (35.83%) | 121 (79.08%) | 32 (20.92%) |  |
| Strongly Agree | 31 (8.40%) | 29 (93.55%) | 2 (6.45%) |  | 65 (15.22%) | 55 (84.62%) | 10 (15.38%) |  |
